# Supplementary material for: Discovery and application of insertion-deletion (INDEL) polymorphisms for QTL mapping of early life-history traits in Atlantic salmon
Source: BMC Genomics. 2010 Mar 8;11:156. doi: 10.1186/1471-2164-11-156 (PMC2838853; doi:10.1186/1471-2164-11-156)
Supplement: Additional file 2 — Information on developed 76 locus single-run INDEL panel in Atlantic salmon. Information on fluorescence labeling, primer concentrations, PCR pooling and links to alignments, INDEL motifs and GENESCAN (Burge and Karlin 1997) predictions of genes/exons are available in html format. [file 1471-2164-11-156-S2.ZIP › Additionalfile2/table4868.html]

Contig Alignment

```
Base	A B C D E F G H I J K L M N O P Q R S T U V W X Y Z   
1	A A A A . . . . . . . . A . . . . . . . . . . . . .    
2	G G G G . . . . G . . . G . . . . . . . . . . . . .    
3	T T T T . . . . T . . . T . . . . . . . . . . . . .    
4	G G G G . . . G G . . . G . . . . . . . . . . . . .    
5	C C C C . . . C C . . . C . . . . . . . . . . . . .    
6	A A A A . . . A A . A . A . . . . . . . . . . . . .    
7	G G G G . . . G G . G . G . . . . . . . . . . . . .    
8	A A A A . . . A A . A . A . . . . . . . . . . . . .    
9	G G G G . . . G G . G . G . . . . . . . . . . . . .    
10	C C C C . . . C C . C . C . . . . . . . . . . . . .    
11	G G G G . . . G G . G . G . . . . . . . . . . . . .    
12	T T T T . . . T T . T . T . . . . . . . . . . . . .    
13	G G G G . . . G G . G . G . . . . . . . . . . . . .    
14	G G G G . . . G G . G . G . . . . . . . . . . . . .    
15	T T T T . . . T T . T . T . . . . . . . . . . . . .    
16	G G G G . . . G G . G . G . . . . . . . . . . . . .    
17	T T T T . . . T T . T . T . . . . . . . . . . . . .    
18	G G G G . . . G G . G . G . . . . . . . . . . . . .    
19	C C C C . . . C C . C . C . . . . . . . . . . . . .    
20	T T T T . . . T T . T . T . . . . . . . . . . . . .    
21	T T T T . . . T T . T . T . . . . . . . . . . . . .    
22	T T T T . . . T T . T . T . . . . . . . . . . . . .    
23	A A A A . . . A A . A . A . . . . . . . . . . . . .    
24	T T T T . . . T T . T . T . . . . . . . . . . . . .    
25	T T T T . . . T T . T . T . . . . . . . . . . . . .    
26	A A A A . . . A A . A . A . . . . . . . . . . . . .    
27	G G G G . . . G G . G . G . . . . . . . . . . . . .    
28	C C C C . . . C C . C . C . . . . . . . . . . . . .    
29	T T T T . . . T T . T . T . . . . . . . . . . . . .    
30	A A A A . . . A A . A . A . . . . . . . . . . . . .    
31	C C C C . . . C C . C . C . . . . . . . . . . . . .    
32	T T T T . . . T T . T . T . . . . . . . . . . . . .    
33	G G G G . . . G G . G . G . . . . . . . . . . . . .    
34	A A A A . . . A A . A . A . . . . . . . . . . . . .    
35	T T T T . . . T T . T . T . . . . . . . . . . . . .    
36	T T T T . . . T T . T . T . . . . . . . . . . . . .    
37	A A A A . . . A A . A . A . . . . . . . . . . . . .    
38	A A A A . . . A A . A . A . . . . . . . . . . . . .    
39	A A A A . . . A A . A . A . . . . . . . . . . . . .    
40	G G G G . . . G G . G . G . . . . . . . . . . . . .    
41	G G G G . . . G G . G . G . . . . . . . . . . . . .    
42	T T T T . . . T T . T . T . . . . . . . . . . . . .    
43	A A A A . . . A A . A . A . . . . . . . . . . . . .    
44	T T T T . . . T T . T . T . . . . . . . . . . . . .    
45	G G G G . . . G G . G . G . . . . . . . . . . . . .    
46	A A A A . . . A A . A . A . . . . . . . . . . . . .    
47	C C C C . . . C C . C . C . . . . . . . . . . . . .    
48	C C C C . . . C C . C . C . . . . . . . . . . . . .    
49	T T T T T T . T T T T . T T T . T T . . . . . . . .    
50	C C C C C C . C C C C . C C C . C C . . . . . . . .    
51	C C C C C C . C C C C . C C C . C C . . . . . . . .    
52	T T T T T T . T T T T . T T T . T T . . . . . . . .    
53	T T T T T T . T T T T . T T T . T T . . . . . . . .    
54	G G G G G G . G G G G . G G G . G G . . . . . . . .    
55	A A A A A A . A A A A . A A A . A A . . . . . . . .    
56	C C C C C C . C C C C . C C C C C C . . . . . . . .    
57	C C C C C C . C C C C . C C C C C C . . . . . . . .    
58	C C C C C C . C C C C . C C C C C C . . . . . . . .    
59	C C C C C C . C C C C . C C C C C C . . . . . . . .    
60	T T T T T T . T T T T . T T T T T T . . . . . . . .    
61	A A A A A A . A A A A . A A A A A A . . . . . . . .    
62	G G G G G G . G G G G . G G G G G G . . . . . . . .    
63	A A A A A A . A A A A . A A A A A A . . . . . . . .    
64	C C C C C C . C C C C . C C C C C C . . . . . . . .    
65	C C C C C C . C C C C . C C C C C C . . . . . . . .    
66	T T T T T T . T T T T . T T T T T T . . . . . . . .    
67	A A A A A A . A A A A . A A A A A A . . . . . . . .    
68	G G G G G G . G G G G . G G G G G G . . . . . . . .    
69	G G G G G G . G G G G . G G G G G G . . . . . . . .    
70	T T T T T T . T T T T . T T T T T T . . . . . . . .    
71	A A A A A A . A A A A . A A A A A A . . . . . . . .    
72	A A A A A A . A A A A . A A A A A A A . . . . . . .    
73	T T T T T T . T T T T . T T T T T T T . . . . . . .    
74	T T T T T T . T T T T . T T T T T T T . . . . . . .    
75	G G G G G G . G G G G . G G G G G G G . . . . . . .    
76	G G G G G G . G G G G . G G G G G G G . . . . . . .    
77	A A A A A A . A A A A . A A A A A A A . . . . . . .    
78	A A A A A A . A A A A . A A A A A A A . . . . . . .    
79	A G G G G G . G G G G . G G G G G G G . . . . . . .    
80	A A A A A A . A A A A . A A A A A A A . . . . . . .    
81	A A A A A A . A A A A . A A A A A A A . . . . . . .    
82	A A A A A A . A A A A . A A A A A A A . . . . . . .    
83	C C C C C C . C C C C . C C C C C C C . . . . . . .    
84	A A A A A A . A A A A . A A A A A A A . . . . . . .    
85	A A A A A A A A A A A . A A A A A A A . . . . . . .    
86	A A A A A A A A A A A . A A A A A A A . . . . . . .    
87	A A A A A A A A A A A . A A A A A A A . . . . . . .    
88	C C C C C C C C C C C . C C C C C C C . . . . . . .    
89	A A A A A A A A A A A . A A A A A A A . . . . . . .    
90	C C C C C C C C C C C . C C C C C C C . . . . . . .    
91	A A A A A A A A A A A . A A A A A A A . . . . . . .    
92	C C C C C C C C C C C . C C C C C C C . . . . . . .    
93	A A A A A A A A A A A . A A A A A A A . . . . . . .    
94	T T T T T T T T T T T . T T T T T T T . . . . . . .    
95	G G G G G G G G G G G . G G G G G G G . . . . . . .    
96	C C C C C C C C C C C . C C C C C C C . . . . . . .    
97	A A A A A A A A A A A . A A A A A A A . . . . . . .    
98	A A A A A A A A A A A . A A A A A A A . . . . . . .    
99	C C C C C C C C C C C . C C C C C C C . . . . . . .    
100	T T T T T T T T T T T . T T T T T T T . . . . . . .    
101	A A A A A A A A A A A . A A A A A A A . . . . . . .    
102	G G G G G G G G G G G . G G G G G G G . . . . . . .    
103	T T T T T T T T T T T . T T T T T T T . . . . . . .    
104	G G G G G G G G G G G . G G G G G G G . . . . . . .    
105	G G G G G G G G G G G . G G G G G G G . . . . . . .    
106	C C C C C C C C C C C . C C C C C C C . . . . . . .    
107	C C C C C C C C C C C . C C C C C C C . . . . . . .    
108	A A A A A A A A A A A . A A A A A A A . . . . . . .    
109	G G G G G G G G G G G . G G G G G G G . . . . . . .    
110	C C C C C C C C C C C . C C C C C C C . . . . . . .    
111	C C C C C C C C C C C . C C C C C C C . . . . . . .    
112	T T T T T T T T T T T . T T T T T T T . . . . . . .    
113	T T T T T T T T T T T . T T T T T T T . . . . . . .    
114	C C C C C C C C C C C . C C C C C C C . . . . . . .    
115	A A A A A A A A A A A . A A A A A A A . . . . . . .    
116	G G G G A A G G G G G . G G G G G G G . . . . . . .    
117	G G G G G G G G G G G . G G G G G G G . . . . . . .    
118	A A A A A A A A A A A . A A A A A A A . . . . . . .    
119	G G G G G G G G G G G . G G G G G G G . . . . . . .    
120	G G G G G G G G G G G . G G G G G G G . . . . . . .    
121	G G G G G G G G G G G . G A G G G G G . . . . . . .    
122	A A A A A A A A A A A . A A A A A A A . . . . . . .    
123	A A A A A A A A A A A . A A A A A A A . . . . . . .    
124	G G G G G G G G G G G . G G G G G G G . . . . . . .    
125	A A A A A A A A A A A . A A A A A A A . . . . . . .    
126	G G G G G G G G G G G . G G G G G G G . . . . . . .    
127	G G G G G G G G G G G . G G G G G G G . . . . . . .    
128	C C C C C C C C C C C . C C C C C C C . . . . . . .    
129	T T T T T T T T T T T . T T T T T T T . . . . . . .    
130	C C C C C C C C C C C . C C C C C C C . . . . . . .    
131	C C C C C C C C C C C . C C C C C C C . . . . . . .    
132	G G G G G G G G G G G . G G G G G G G . . . . . . .    
133	C C C C C C C C C C C . C C C C C C C . . . . . . .    
134	T T T T T T T T T T T . T T T T T T T . . . . . . .    
135	T T T T T T T T T T T . T T T T T T T . . . . . . .    
136	T T T T T T T T T T T . T T T T T T T . . . . . . .    
137	T T T T T T T T T T T . T T T T T T T . . . . . . .    
138	A A A A A A A A A A A . A A A A A A A . . . . . . .    
139	C C C C C C C C C C C . C C C C C C C . . . . . . .    
140	A A A A A A A A A A A . A A A A A A A . . . . . . .    
141	A A A A A A A A A A A . A A A A A A A . . . . . . .    
142	C C C C C C C C C C C . C C C C C C C . . . . . . .    
143	T T T T T T T T T T T . T T T T T T T . . . . . . .    
144	T T T T T T T T T T T . T T T T T T T . . . . . . .    
145	C C C C C C C C C C C . C C C C C C C . . . . . . .    
146	A A A A A A A A A A A . A A A A A A A . . . . . . .    
147	A G G G G G G G G G G . G G G G G G G . . . . . . .    
148	A A A A A A A A A A A . A A A A A A A . . . . . . .    
149	A A A A A A A A A A A . A A A A A A A . . . . . . .    
150	C C C C C C C C C C C . C C C C C C C . . . . . . .    
151	G G G G G G G G G G G . G G G G G G G . . . . . . .    
152	C C C C C C C C C C C . C C C C C C C . . . . . . .    
153	A A A A A A A A A A A . A A A A A A A . . . . . . .    
154	C C C C C C C C C C C . C C C C C C C . . . . . . .    
155	A A A A A A A A A A A . A A A A A A A . . . . . . .    
156	A A A A A A A A A A A . A A A A A A A . . . . . . .    
157	C C C C C C C C C C C . C C C C C C C . . . . . . .    
158	T T T T T T T T T T T . T T T T T T T . . . . . . .    
159	C C C C C C C C C C C . C C C C C C C . . . . . . .    
160	C C C C C C C C C C C . C C C C C C C . . . . . . .    
161	T T T T T T T T T T T . T T T T T T T . . . . . . .    
162	C C C C C C C C C C C . C C C C C C C . . . . . . .    
163	A A A A A A A A A A A . A A A A A A A . . . . . . .    
164	A A A A A A A A A A A . A A A A A A A . . . . . . .    
165	A A A A A A A A A A A . A A A A A A A . . . . . . .    
166	C C C C C C C C C C C . C C C C C C C . . . . . . .    
167	T T T T T T T T T T T . T T T T T T T . . . . . . .    
168	G G G G G G G G G G G . G G G G G G G . . . . . . .    
169	G G G G G G G G G G G . G G G G G G G . . . . . . .    
170	G G G G G G G G G G G . G G G G G G G . . . . . . .    
171	G G G G G G G G G G G . G G G G G G G . . . . . . .    
172	T T T T T T T T T T T . T T T T T T T . . . . . . .    
173	G G G G G G G G G G G . G G G G G G G . . . . . . .    
174	T T T T T T T T T T T . T T T T T T T . . . . . . .    
175	G G G G G G G G G G G . G G G G G G G . . . . . . .    
176	A A A A A A A A A A A . A A A A A A A . . . . . . .    
177	A A A A A A A A A A A . A A A A A A A . . . . . . .    
178	A A A A A A A A A A A . A A A A A A A . . . . . . .    
179	C C C C C C C C C C C . C C C C C C C . . . . . . .    
180	G G G G G G G G G G G . G G G G G G G . . . . . . .    
181	C C C C C C C C C C C . C C C C C C C . . . . . . .    
182	T T T T T T T T T T T . T T T T T T T . . . . . . .    
183	C C C C C C C C C C C . C C C C C C C . . . . . . .    
184	T T T T T T T T T T T . T T T T T T T . . . . . . .    
185	G G G G G G G G G G G . G G G G G G G . . . . . . .    
186	A A A A A A A A A A A . A A A A A A A . . . . . . .    
187	A A A A A A A A A A A . A A A A A A A . . . . . . .    
188	C C C C C C C C C C C . C C C C C C C . . . . . . .    
189	C C C C C C C C C C C . C C C C C C C . . . . . . .    
190	T T T T T T T T T T T . T T T T T T T . . . . . . .    
191	T T T T T T T T T T T . T T T T T T T . . . . . . .    
192	G G G G G G G G G G G . G G G G G G G . . . . . . .    
193	T T T T T T T T T T T . T T T T T T T . . . . . . .    
194	A A A A A A A A A A A . A A A A A A A . . . . . . .    
195	G G G G G G G G G G G . G G G G G G G . . . . . . .    
196	A A A A A A A A A A A . A A A A A A A . A . . . . .    
197	T T T T T T T T T T T . T T T T T T T . T . . . . .    
198	G G G G G G G G G G G . G G G G G G G . G . . . . .    
199	G G G G G G G G G G G . G G G G G G G . G . . . . .    
200	A A A A A A A A A A A . A A A A A A A . A . . . . .    
201	A A A A A A A A A A A . A A A A A A A . A . . . . .    
202	A A A A A A A A A A A . A A A A A A A . A . . . . .    
203	T T T T T T T T T T T . T T T T T T T . T . . . . .    
204	A A A A A A A A A A A . A A A A A A A . A . . . . .    
205	C C C C C C C C C C C C C C C C C C C . C . . . . .    
206	A A A A A A A A A A A A A A A A A A A . A . . . . .    
207	G G G G G G G G G G G G G G G G G G G . G . . . . .    
208	T T T T T T T T T T T T T T T T T T T . T . . . . .    
209	G G G G G G G G G G G G G G G G G G G . G . . . . .    
210	A A A A A A A A A A A A A A A A A A A . A . . . . .    
211	A A A A A A A A A A A A A A A A A A A . A . . . . .    
212	T T T T T T T T T T T T T T T T T T T . T . . . . .    
213	A A A A A A A A A A A A A A A A A A A . A . . . . .    
214	G G G G G G G G G G G G G G G G G G G . G . . . . .    
215	A A A A A A A A A A A A A A A A A A A . A . . . . .    
216	G G G G G G G G G G G G G G G G G G G . G . . . . .    
217	C C C C C C C C C C C C C C C C C C C . C . . . . .    
218	T T T T T T T T T T T T T T T T T T T . T . . . . .    
219	G G G G G G G G G G G G G G G G G G G . G . . . . .    
220	A A A A A A A A A A A A A A A A A A A . A . . . . .    
221	C C C C C C C C C C C C C C C C C C C . C . . . . .    
222	A A A A A A A A A A A A A A A A A A A . A . . . . .    
223	C C C C C C C C C C C C C C C C C C C . C . . . . .    
224	G G G G G G G G G G G G G G G G G G G . G . . . . .    
225	G G G G G G G G G G G G G G G G G G G . G . . . . .    
226	T T T T T T T T T T T T T T T T T T T . T . . . . .    
227	T T T T T T T T T T T T T T T T T T T . T . . . . .    
228	C C C C C C C C C C C C C C C C C C C . C . . . . .    
229	C C C C C C C C C C C C C C C C C C C . C . . . . .    
230	C C C C C C C C C C C C C C C C C C C . C . . . . .    
231	T T T T T T T T T T T T T T T T T T T . T . . . . .    
232	A A A A A A A A A A A A A A A A A A A . A . . . . .    
233	T T T T T T T T T T T T T T T T T T T . T . . . . .    
234	T T T T T T T T T T T T T T T T T T T . T . . . . .    
235	C C C C C C C C C C C C C C C C C C C . C . . . . .    
236	T T T T T T T T T T T T T T T T T T T . T . . . . .    
237	C C C C C C C C C C C C C C C C C C C . C . . . . .    
238	T T T T T T T T T T T T T T T T T T T . T . . . . .    
239	G G G G G G G G G G G G G G G G G G G . G . . . . .    
240	A A A A A A A A A A A A A A A A A A A . A . . . . .    
241	T T T T T T T T T T T T T T T T T T T . T . . . . .    
242	A A A A A A A A A A A A A A A A A A A . A . . . . .    
243	A A A A A A A A A A A A A A A A A A A . A . . . . .    
244	A A A A A A A A A A A A A A A A A A A . A . . . . .    
245	C C C C C C C C C C C C C C C C C C C . C . . . . .    
246	A A A A A A - A A A A - A A - A - - - . A . . . . .  6	 indel  
247	A A A A A A - A A A A - A A - A - - - . A . . . . .  6	 indel  
248	A A A A A A - A A A A - A A - A - - - . A . . . . .  6	 indel  
249	C C C C C C - C C C C - C C - C - - - . C . . . . .  6	 indel  
250	T T T T T T T T T T T T T T T T T T T . T . . . . .    
251	G G G G G G G G G G G G G G G G G G G . G . . . . .    
252	T T T T T T T T T T T T T T T T T T T . T . . . . .    
253	T T T T T T T T T T T T T T T T T T T . T . . . . .    
254	C C C C C C C C C C C C C C C C C C C . C . . . . .    
255	T T T T T T T T T T T T T T T T T T T . T . . . . .    
256	A A A A A A A A A A A A A A A A A A A . A . . . . .    
257	C C C C C C C C C C C C C C C C C C C . C . . . . .    
258	A A A A A A A A A A A A A A A A A A A . A . . . . .    
259	C C C C C C C C C C C C C C C C C C C . C . . . . .    
260	A A A A A A A A A A A A A A A A A A A . A . . . . .    
261	A A A A A A A A A A A A A A A A A A A . A . . . . .    
262	T T T T T T T T T T T T T T T T T T T . T . . . . .    
263	A A A A A A A A A A A A A A A A A A A . A . . . . .    
264	C C C C C C C C C C C C C C C C C C C . C . . . . .    
265	A A A A A A A A A A A A A A A A A A A . A . . . . .    
266	T T T T T T T T T T T T T T T T T T T . T . . . . .    
267	T T T T T T T T T T T T T T T T T T T . T . . . . .    
268	T T T T T T T T T T T T T T T T T T T . T . . . . .    
269	C C C C C C C C C C C C C C C C C C C . C . . . . .    
270	T T T T T T T T T T T T T T T T T T T . T . . . . .    
271	A A A A A A A A A A A A A A A A A A A . A . . . . .    
272	T T T T T T T T T T T T T T T T T T T . T . . . . .    
273	C C C C C C C C C C C C C C C C C C C . C . . . . .    
274	T T T T T T T T T T T T T T T T T T T . T . . . . .    
275	G G G G G G G G G G G G G G G G G G G . G . . . . .    
276	A A A A A A A A A A A A A A A A A A A . A . . . . .    
277	A A A A A A A A A A A A A A A A A A A . A . . . . .    
278	C C C C C C C C C C C C C C C C C C C . C . . . . .    
279	T T T T T T T T T T T T T T T T T T T . T . . . . .    
280	T T T T T T T T T T T T T T T T T T T . T . . . . .    
281	T T T T T T T T T T T T T T T T T T T . T . . . . .    
282	C C C C C C C C C C C C C C C C C C C . C . . . . .    
283	T T T T T T T T T T T T T T T T T T T . T . . . . .    
284	G G G G G G G G G G G G G G G G G G G . G . . . . .    
285	T T T T T T T T T T T T T T T T T T T . T . . . . .    
286	A A A A A A A A A A A A A A A A A A A . A . . . . .    
287	A A A A A A A A A A A A A A A A A A A . A . . . . .    
288	T T T T T T T T T T T T T T T T T T T . T . . . . .    
289	G G G G G G G G G G G G G G G G G G G . G . . . . .    
290	T T T T T T T T T T T T T T T T T T T . T . . . . .    
291	T T T T T T T T T T T T T T T T T T T T T . . . . .    
292	A A A A A A A A A A A A A A A A A A A A A . . . . .    
293	C C C C C C C C C C C C C C C C C C C C C . . . . .    
294	A A A A A A A A A A A A A A A A A A A A A . . . . .    
295	T T T T T T T T T T T T T T T T T T T T T . . . . .    
296	C C C C C C C C C C C C C C C C C C C C C . . . . .    
297	C C C C C C C C C C C C C C C C C C C C C . . . . .    
298	T T T T T T T T T T T T T T T T T T T T T . . . . .    
299	G G G G G G G G G G G G G G G G G G G G G . . . . .    
300	A A A A A A A A A A A A A A A A A A A A A . . . . .    
301	A A A A A A A A A A A A A A A A A A A A A . . . . .    
302	C C C C C C C C C C C C C C C C C C C C C . . . . .    
303	A A A A A A A A A A A A A A A A A A A A A . . . . .    
304	G G G G G G G G G G G G G G G G G G G G G . . . . .    
305	A A A A A A A A A A A A A A A A A A A A A . . . . .    
306	C C C C C C C C C C C C C C C C C C C C C . . . . .    
307	C C C C C C C C C C C C C C C C C C C C C . . . . .    
308	C C C C C C C C C C C C C C C C C C C C C . . . . .    
309	C C C C C C C C C C C C C C C C C C C C C . . . . .    
310	T T T T T T T T T T T T T T T T T T T T T . . . . .    
311	G G G G G G G G G G G G G G G G G G G G G . . . . .    
312	G G G G G G G G G G G G G G G G G G G G G . . . . .    
313	T T T T T T T T T T T T T T T T T T T T T . . . . .    
314	T T T T T T T T T T T T T T T T T T T T T . . . . .    
315	T T T T T T T T T T T T T T T T T T T T T . . . . .    
316	A A A A A A A A A A A A A A A A A A A A A . . . . .    
317	A A A A A A A A A A A A A A A A A A A A A . . . . .    
318	G G G G G G G G G G G G G G G G G G G G G . . . . .    
319	G G G G G G G G G G G G G G G G G G G G G . . . . .    
320	T T T T T T T T T T T T T T T T T T T T T . . . . .    
321	T T T T T T T T T T T T T T T T T T T T T . . . . .    
322	A A A A A A A A A A A A A A A A A A A A A . . . . .    
323	G G G G G G G G G G G G G G G G G G G G G . . . . .    
324	A A A A A A A A A A A A A A A A A A A A A . . . . .    
325	T T T T T T T T T T T T T T T T T T T T T . . . . .    
326	C C C C C C C C C C C C C C C C C C C C C . . . . .    
327	A A A A A A A A A A A A A A A A A A A A A . . . . .    
328	T T T T T T T T T T T T T T T T T T T T T . . . . .    
329	T T T T T T T T T T T T T T T T T T T T T . . . . .    
330	G G G G G G G G G G G G G G G G G G G G G . . . . .    
331	A A A A A A A A A A A A A A A A A A A A A . . . . .    
332	T T T T T T T T T T T T T T T T T T T T T . . . . .    
333	G G G G G G G G G G G G G G G G G G G G G . . . . .    
334	C C C C C C C C C C C C C C C C C C C C C . . . . .    
335	A A A A A A A A A A A A A A A A A A A A A . . . . .    
336	A A A A A A A A A A A A A A A A A A A A A . . . . .    
337	T T T T T T T T T T T T T T T T T T T T T . . . . .    
338	G G G G G G G G G G G G G G G G G G G G G . . . . .    
339	G G G G G G G G G G G G G G G G G G G G G . . . . .    
340	C C C C C C C C C C C C C C C C C C C C C . . . . .    
341	T T T T T T T T T T T T T T T T T T T T T . . . . .    
342	G G G G G G G G G G G G G G G G G G G G G . G . . .    
343	A A A A A A A A A A A A A A A A A A A A A . A . . .    
344	T T T T T T T T T T T T T T T T T T T T T . T . . .    
345	G G G G G G G G G G G G G G G G G G G G G . G . . .    
346	T T T T T T T T T T T T T T T T T T T T T . T . . .    
347	C C C C C C C C C C C C C C C C C C C C C . C . . .    
348	T T T T T T T T T T T T T T T T T T T T T . T . . .    
349	C C C C C C C C C C C C C C C C C C C C C . C . . .    
350	G G G G G G G G G G G G G G G G G G G G G . G . . .    
351	G G G G G G G G G G G G G G G G G G G G G . G . . .    
352	T T T T T T T T T T T T T T T T T T T T T . T . . .    
353	A A A A A A A A A A A A A A A A A A A A A . A . . .    
354	A A A A A A A A A A A A A A A A A A A A A . A . . .    
355	G G G G G G G G G G G G G G G G G G G G G . G . . .    
356	G G G G G G G G G G G G G G G G G G G G G . G . . .    
357	T T T T T T T T T T T T T T T T T T T T T . T . . .    
358	T T T T T T T T T T T T T T T T T T T T T . T . . .    
359	A A A A A A A A A A A A A A A A A A A A A . A . . .    
360	C C C C C C C C C C C C C C C C C C C C C . C . . .    
361	A A A A A A A A A A A A A A A A A A A A A . A . . .    
362	C C C C C C C C C C C C C C C C C C C C C . C . . .    
363	A A A A A A A A A A A A A A A A A A A A A . A . . .    
364	T T T T T T T T T T T T T T T T T T T T T . T . . .    
365	C C C C C C C C C C C C C C C C C C C C C . C . . .    
366	T T T T T T T T T T T T T T T T T T T T T . T . . .    
367	T T T T T T T T T T T T T T T T T T T T T . T . . .    
368	A A A A A A A A A A A A A A A A A A A A A . A . . .    
369	A G G G G G G G G G G G G G G G G G G G G . G . . .    
370	. T T T T T T T T T T T T T T T T T T T T . T . . .    
371	. A A A A A A A A A A A A A A A A A A A A . A . . .    
372	. T T T T T T T T T T T T T T T T T T T T . T . . .    
373	. T T T T T T T T T T T T T T T T T T T T . T . . .    
374	. C C C C C C C C C C C C C C C C C C C C . C . . .    
375	. T T T T T T T T T T T T T T T T T T T T . T . . .    
376	. T T T T T T T T T T T T T T T T T T T T . T . . .    
377	. T T T T T T T T T T T T T T T T T T T T . T . . .    
378	. T T T T T T T T T T T T T T T T T T T T . T . . .    
379	. C C C C C C C C C C C C C C C C C C C C . C . . .    
380	. T T T T T T T T T T A T T T T T T T T T . T . . .    
381	. T T T T T T T T T T T T T T T T T T T T . T . . .    
382	. T T T T T T T T T T T T T T T T T T T T . T . . .    
383	. C C C C C C C C C C C C C C C C C C C C . C . . .    
384	. A A A A A A A A A A A A A A A A A A A A . A . . .    
385	. - - - - - - - - - - A - - - - - - - - - . - . . .    
386	. T T T T T T T T T T T T T T T T T T T T . T . . .    
387	. C C C C C C C C C C C C C C C C C C C C . C . . .    
388	. A A A A A A A A A A A A A A A A A A A A . A . . .    
389	. A A A A A A A A A A N A A A A A A A A A . A . . .    
390	. C C C C C C C C C C C C C C C C C C C C . C . . .    
391	. C C C C C C C C C C C C C C C C C C C C . C . . .    
392	. C C C C C C C C C C C C C C C C C C C C . C . . .    
393	. C C C C C C C C C C C C C C C C C C C C . C . . .    
394	. T T T T T T T T T T T T T T T T T T T T . T . . .    
395	. T T T T T T T T T T T T T T T T T T T T . T . . .    
396	. G G G G G G G G G G G G G G G G G G G G . G . . .    
397	. T T T T T T T T T T T T T T T T T T T T . T . . .    
398	. A A A A A A A A A A A A A A A A A A A A . A . . .    
399	. G G G G G G G G G G G G G G G G G G G G . G . . .    
400	. A A A A A A A A A A A A A A A A A A A A . A . . .    
401	. C C C C C C C C C C C C C C C C C C C C . C . . .    
402	. G G G G G G G G G G G G G G G G G G G G . G . . .    
403	. T T T T T T T T T T T T T T T T T T T T . T . . .    
404	. A A A A A A A A A A A A A A A A A A A A . A . . .    
405	. G G G G G G G G G G G G G G G G G G G G . G . . .    
406	. G G G G G G G G G G G G G G G G G G G G . G . . .    
407	. T T T T T T T T T T T T T T T T T T T T . T . . .    
408	. G G G G G G G G G G G G G G G G G G G G . G . . .    
409	. A A A A A A A A A A A A A A A A A A A A . A . . .    
410	. C C C C C C C C C C C C C C C C C C C C . C . . .    
411	. A A A A A A A A A A A A A A A A A A A A . A . . .    
412	. A A A A A A A A A A A A A A A A A A A A . A . . .    
413	. T T T T T T T T T T T T T T T T T T T T . T . . .    
414	. A A A A A A A A A A A A A A A A A A A A . A . . .    
415	. C C C C C C C C C C C C C C C C C C C C . C . . .    
416	. A A A A A A A A A A A A A A A A A A A A . A . . .    
417	. A A A A A A A A A A A A A A A A A A A A . A . . .    
418	. C C C C C C C C C C C C C C C C C C C C . C . . .    
419	. T T T T T T T T T T T T T T T T T T T T . T . . .    
420	. T T T T T T T T T T T T T T T T T T T T . T . . .    
421	. T T T T T T T T T T T T T T T T T T T T . T . . .    
422	. A A A A A A A A A A A A A A A A A A A A . A . . .    
423	. T T T T T T T T T T T T T T T T T T T T . T . . .    
424	. T T T T T T T T T T T T T T T T T T T T . T . . .    
425	. C C C C C C C C C C C C C C C C C C C C . C . . .    
426	. T T T T T T T T T T T T T T T T T T T T . T . . .    
427	. C C C C C C C C C C C C C C C C C C C C . C . . .    
428	. T T T T T T T T T T A T T T T T T T T T . T . . .    
429	. C C C C C C C C C C C C C C C C C C C C . C . . .    
430	. C C C C C C C C C C C C C C C C C C C C . C . . .    
431	. G G G G G G G G G G G G G G G G G G G G . G . . .    
432	. T T T T T T T T T T T T T T T T T T T T . T . . .    
433	. A A A A A A G A A A A A A A G A A A A A . A . . .    
434	. C C C C C C C C C C C C C C C C C C C C . C . . .    
435	. G G G G G G G G G G G G G G G G G G G G . G . . .    
436	. T T T T T T T T T T T T T T T T T T T T . T . . .    
437	. T T T T T T T T T T T T T T T T T T T T . T . . .    
438	. C C C C C C C C C C C C C C C C C C C C . C . . .    
439	. A A A A A A A A A A A A A A A A A A A A . A . . .    
440	. . C C C C C C C C C C C C C C C C C C C . C . . .    
441	. . T T T T T T T T T T T T T T T T T T T . T . . .    
442	. . T T T T T T T T T T T T T T T T T T T . T . . .    
443	. . T T T T T T T T T T T T T T T T T T T . T . . .    
444	. . T T T T T T T T T T T T T T T T T T T . T . . .    
445	. . A A A A A A A A A A A A A A A A A A A . A . . .    
446	. . T T T T T T T T T T T T T T T T T T T . T . . .    
447	. . A A A A A A A A A A A A A A A A A A A . A . . .    
448	. . T T T T T T C T T T T T T T T T T T T . T . . .    
449	. . C C C C C C C C C C C C C C C C C C C . C . . .    
450	. . A A A A A A A A A A A A A A A A A A A . A . . .    
451	. . C C C C C C C C C C C C C C C C C C C . C . . .    
452	. . A A A A A A A A A A A A A A A A A A A . A . . .    
453	. . G G G G G G G G G G G G G G G G G G G . G . . .    
454	. . G G G G G G G G G G G G G G G G G G G . G . . .    
455	. . A A A A A A A A A A A A A A A A A A A . A . . .    
456	. . A A A A A A A A A A A A A A A A A A A . A . . .    
457	. . A A A A A A A A A A A A A A A A A A A . A . . .    
458	. . G G G G G G G G G G G G G G G G G G G . G . . .    
459	. . T T T T T T T T T T T T T T T T T T T . T . . .    
460	. . T T T T T T T T T T T T T T T T T T T . T . . .    
461	. . T T T T T T T T T T T T T T T T T T T . T . . .    
462	. . G G G G G G G G G G G G G G G G G G G . G . . .    
463	. . A A A A A A A A A A A A A A A A A A A . A . . .    
464	. . A A A A A A A A A A A A A A A A A A A . A . . .    
465	. . T T T T T T T T T T T T T T T T T T T . T . . .    
466	. . T T T T T T T T T T T T T T T T T T T . T . . .    
467	. . C C G G G G G G G G G G G G G G G G G . G . . .    
468	. . . . C C C C C C C C C C C C C C C C C . C C . .    
469	. . . . A A A A A A A A A A A A A A A A A . A A . .    
470	. . . . T T T T T T T T T T T T T T T T T . T T . .    
471	. . . . G G G G G G G G G G G G G G G G G . G G . .    
472	. . . . T T T T T T T T T T T T T T T T T . T T . .    
473	. . . . A A A A A A A A A A A A A A A A A . A A . .    
474	. . . . G G G G G G G G G G G G G G G G G . G G . .    
475	. . . . C C C C C C C C C C C C C C C C C . C C . .    
476	. . . . C C C C C C C C C C C C C C C C C . C C . .    
477	. . . . C C C C C C C C C C C C C C C C C . C C . .    
478	. . . . A A A A A A A A A A A A A A A A A . A A . .    
479	. . . . T T T T T T T T T T T T T T T T T . T T . .    
480	. . . . C C C C C C C C C C C C C C C C C . C C . .    
481	. . . . T T T T T T T T T T T T T T T T T . T T . .    
482	. . . . C C C C C C C C C C C C C C C C C . C C . .    
483	. . . . T T T T T T T T T T T T T T T T T . T T . .    
484	. . . . T T T T T T T T T T T T T T T T T . T T . .    
485	. . . . G G G G G G G G G G G G G G G G G . G G . .    
486	. . . . T T T T T T T T T T T T T T T T T . T T . .    
487	. . . . G G G G G G G G G G G G G G G G G . G G . .    
488	. . . . T T T T T T T T T T T T T T T T T . T T . .    
489	. . . . G G G G G G G G G G G G G G G G G . G G . .    
490	. . . . A A A A A A A A A A A A A A A A A . A A . .    
491	. . . . A A A A A A A A A A A A A A A A A . A A . .    
492	. . . . A A A A A A A A A A A A A A A A A . A A . .    
493	. . . . G G G G G G G G G G G G G G G G G . G G . .    
494	. . . . T T T T T T T T T T T T T T T T T . T T . .    
495	. . . . C C A A A A A A A A A A A A A A A . A A . .    
496	. . . . . . A A A A A A A A A A A A A A A . A A . .    
497	. . . . . . A A A A A A A A A A A A A A A . A A . .    
498	. . . . . . A A A A A A A A A A A A A A A . A A . .    
499	. . . . . . T T T T T T T T T T T T T T T . T T . .    
500	. . . . . . A A A A A A A A A A A A A A A . A A . .    
501	. . . . . . T T T T T T T T T T T T T T T . T T . .    
502	. . . . . . T T T T T T T T T T T T T T T . T T . .    
503	. . . . . . C C C C C C C C C C C C C C C . C C . .    
504	. . . . . . T T T T T T T T T T T T T T T . T T . .    
505	. . . . . . T T T T T T T T T T T T T T T . T T . .    
506	. . . . . . A A A A A A A A A A A A A A A . A A . .    
507	. . . . . . A A A A A A A A A A A A A A A . A A . .    
508	. . . . . . A A A A A A A A A A A A A A A . A A . .    
509	. . . . . . G G G G G G G G G G G G G G G . G G . .    
510	. . . . . . C C C C C C C C C C C C C C C . C C . .    
511	. . . . . . T T T T T T T T T T T T T T T . T T . .    
512	. . . . . . T T T T T T T T T T T T T T T . T T . .    
513	. . . . . . T T T T T T T T T T T T T T T . T T . .    
514	. . . . . . A A A A A A A A A A A A A A A . A A . .    
515	. . . . . . A A A A A A A A A A A A A A A . A A . .    
516	. . . . . . A A A A A A A A A A A A A A A . A A . .    
517	. . . . . . G G G G G G G G G G G G G G G . G G . .    
518	. . . . . . T T T T T T T T T T T T T T T . T T . .    
519	. . . . . . T T T T T T T T T T T T T T T . T T . .    
520	. . . . . . A A A A A A A A A A A A A A A . A A . .    
521	. . . . . . A A A A A N A A A A A A A A A . A A . .    
522	. . . . . . C C C C C C C C C C C C C C C . C C . .    
523	. . . . . . T T T T T T T T T T T T T T T . T T . .    
524	. . . . . . G G G G G G G G G G G G G G G . G G . .    
525	. . . . . . A A A A A A A A A A A A A A A . A A . .    
526	. . . . . . T T T T T T T T T T T T T T T . T T . .    
527	. . . . . . A A A A A A A A A A A A A A A . A A . .    
528	. . . . . . A A A A A A A A A A A A A A A . A A . .    
529	. . . . . . A A A A A A A A A A A A A A A . A A . .    
530	. . . . . . T T T T T T T T T T T T T T T . T T . .    
531	. . . . . . A A A A A A A A A A A A A A A . A A . .    
532	. . . . . . C C C C C C C C C C C C C C C . C C . .    
533	. . . . . . A A A A A A A A A A A A A A A . A A . .    
534	. . . . . . T T T T T T T T T T T T T T T . T T . .    
535	. . . . . . T T T T T T T T T T T T T T T . T T . .    
536	. . . . . . T T T T T T T T T T T T T T T . T T . .    
537	. . . . . . G G G G G G G G G G G G G G G . G G . .    
538	. . . . . . C C C C C C C C C C C C C C C . C C . .    
539	. . . . . . C C C C C C C C C C C C C C C . C C . .    
540	. . . . . . T T T T T T T T T T T T T T T . T T . .    
541	. . . . . . G G G G G G G G G G G G G G G . G G . .    
542	. . . . . . C C C C C C C C C C C C C C C . C C . .    
543	. . . . . . A A A A A A A A A A A A A A A . A A . .    
544	. . . . . . A A A A A A A A A A A A A A A . A A . .    
545	. . . . . . T T T T T T T T T T T T T T T . T T . .    
546	. . . . . . T T T T T T T T T T T T T T T . T T . .    
547	. . . . . . T T T T T T T T T T T T T T T . T T . .    
548	. . . . . . A A A A A A A A A A A A A A A . A A . .    
549	. . . . . . C C C C C C C C C C C C C C C . C C . .    
550	. . . . . . A A A A A A A A A A A A A A A . A A . .    
551	. . . . . . C C C C C C C C C C C C C C C . C C . .    
552	. . . . . . A A A A A A A A A A A A A A A . A A . .    
553	. . . . . . A A A A A A A A A A A A A A A . A A . .    
554	. . . . . . T T T T T T T T T T T T T T T . T T . .    
555	. . . . . . G G G G G G G G G G G G G G G . G G . .    
556	. . . . . . G G G G G G G G G G G G G G G . G G . .    
557	. . . . . . G G G G G G G G G G G G G G G . G G . .    
558	. . . . . . A A A A A A A A A A A A A A A . A A . .    
559	. . . . . . T T T T T T T T T T T T T T T . T T . .    
560	. . . . . . T T T T T T T T T T T T T T T . T T . .    
561	. . . . . . T T T T T T T T T T T T T T T . T T . .    
562	. . . . . . T T T T T T T T T T T T T T T . T T . .    
563	. . . . . . G G G G G G G G G G G G G G G . G G . .    
564	. . . . . . T T T T T T T T T T T T T T T . T T . .    
565	. . . . . . C C C C C C C C C C C C C C C . C C . .    
566	. . . . . . A A A A A A A A A A A A A A A . A A . .    
567	. . . . . . T T T T T T T T T T T T T T T . T T . .    
568	. . . . . . A A A A A A A A A A A A A A A . A A . .    
569	. . . . . . T T T T T T T T T T T T T T T . T T . .    
570	. . . . . . C C C C C C C C C C C C C C C . C C . .    
571	. . . . . . A A A A A A A A A A A A A A A . A A . .    
572	. . . . . . T T T T T T T T T T T T T T T . T T . .    
573	. . . . . . T T T T T T T T T T T T T T T . T T . .    
574	. . . . . . G G G G G G G G G G G G G G G . G G . .    
575	. . . . . . T T T T T T T T T T T T T T T . T T . .    
576	. . . . . . T T T T T T T T T T T T T T T . T T . .    
577	. . . . . . A A A A A A A A A A A A A A A . A A . .    
578	. . . . . . T T T T T T T T T T T T T T T . T T . .    
579	. . . . . . T T T T T T T T T T T T T T T . T T . .    
580	. . . . . . G G G G G G G G G G G G G G G . G G . .    
581	. . . . . . C C C C C C C C C C C C C C C . C C . .    
582	. . . . . . G G G G G G G G G G G G G G G . G G . .    
583	. . . . . . . A A A A A A A A A A A A A A . A A . .    
584	. . . . . . . A A A A A A A A A A A A A A . A A . .    
585	. . . . . . . A A A A A A A A A A A A A A . A A . .    
586	. . . . . . . C C C C C C C C C C C C C C . C C . .    
587	. . . . . . . A A A A A A A A A A A A A A . A A . .    
588	. . . . . . . A A A A A A A A A A A A A A . A A . .    
589	. . . . . . . G G G G G G G G G G G G G G . G G . .    
590	. . . . . . . G G G G G G G G G G G G G G . G G . .    
591	. . . . . . . T T T T T T T T T T T T T T . T T . .    
592	. . . . . . . C C C C C C C C C C C C C C . C C . .    
593	. . . . . . . T T T T T T T T T T T T T T . T T . .    
594	. . . . . . . T T T T T T T T T T T T T T . T T . .    
595	. . . . . . . G G G G G G G G G G G G G G . G G . .    
596	. . . . . . . G G G G G G G G G G G G G G . G G . .    
597	. . . . . . . A A A A A A A A A A A A A A . A A . .    
598	. . . . . . . A A A A A A A A A A A A A A . A A . .    
599	. . . . . . . G G G G G G G G G G G G G G . G G . .    
600	. . . . . . . A A A A A A A A A A A A A A . A A . .    
601	. . . . . . . G G G G T G G T G T T T T G . G G . .  6	 transversion  
602	. . . . . . . G G G G G G G G G G G G G G . G G . .    
603	. . . . . . . T T T T T T T T T T T T T T . T T . .    
604	. . . . . . . T T T T T T T T T T T T T T . T T . .    
605	. . . . . . . G G G G G G G G G G G G G G . G G . .    
606	. . . . . . . A G A A A A A A A A A A A A . A A . .    
607	. . . . . . . A A A A A A A A A A A A A A . A A . .    
608	. . . . . . . C C C C C C C C C C C C C C . C C . .    
609	. . . . . . . A A A A A A A A A A A A A A . A A . .    
610	. . . . . . . T T T T T T T T T T T T T T . T T . .    
611	. . . . . . . T T T T T T T T T T T T T T . T T . .    
612	. . . . . . . C C C C C C C C C C C C C C . C C . .    
613	. . . . . . . G G G G G G G G G G G G G G . G G . .    
614	. . . . . . . A A A A A A A A A A A A A A . A A . .    
615	. . . . . . . C C C C C C C C C C C C C C . C C . .    
616	. . . . . . . A A A A A A A A A A A A A A . A A . .    
617	. . . . . . . G G G G G G G G G G G G G G . G G . .    
618	. . . . . . . T T T T T T T T T T T T T T . T T . .    
619	. . . . . . . A A A A A A A A A A A A A A . A A . .    
620	. . . . . . . G G G G G G G G G G G G G G . G G . .    
621	. . . . . . . T T T T T T T T T T T T T T . T T . .    
622	. . . . . . . G G G G G G G G G G G G G G G G G . G    
623	. . . . . . . T T T T T T T T T T T T T T T T T . T    
624	. . . . . . . G G G G G G G G G G G G G G G G G . G    
625	. . . . . . . G T T T T T T T T T T T T T T T T . T    
626	. . . . . . . . A A A A A A A A A A A A A A A A . A    
627	. . . . . . . . T T T T T T T T T T T T T T T T . T    
628	. . . . . . . . A A A A A A A A A A A A A A A A . A    
629	. . . . . . . . T T T T T T T T T T T T T T T T . T    
630	. . . . . . . . A A A A A A A A A A A A A A A A . A    
631	. . . . . . . . T T T T T T T T T T T T T T T T . T    
632	. . . . . . . . A A A - A A - A - - - - A A A A . A  6	 indel  
633	. . . . . . . . T T T - T T - T - - - - T T T T . T  6	 indel  
634	. . . . . . . . A A A - A A - A - - - - A A A A . A  6	 indel  
635	. . . . . . . . T T T - T T - T - - - - T T T T . T  6	 indel  
636	. . . . . . . . G G G G G G G G G G G G G G G G . G    
637	. . . . . . . . T T T T T T T T T T T T T T T T . T    
638	. . . . . . . . A A A A A A A A A A A A A A A A . A    
639	. . . . . . . . T T T T T T T T T T T T T T T T . T    
640	. . . . . . . . G G G G G G G G G G G G G G G G . G    
641	. . . . . . . . T T T T T T T T T T T T T T T T . T    
642	. . . . . . . . T T T T T T T T T T T T T T T T . T    
643	. . . . . . . . T T T T T T T T T T T T T T T T . T    
644	. . . . . . . . A A A A A A A A A A A A A A A A . A    
645	. . . . . . . . T T T T T T T T T T T T T T T T . T    
646	. . . . . . . . T T T T T T T T T T T T T T T T . T    
647	. . . . . . . . T T T T T T T T T T T T T T T T . T    
648	. . . . . . . . C C C C C C C C C C C C C C C C . C    
649	. . . . . . . . T T T T T T T T T T T T T T T T . T    
650	. . . . . . . . A A A A A A A A A A A A A A A A . A    
651	. . . . . . . . C C C C C C C C C C C C C C C C . C    
652	. . . . . . . . G G G G G G G G G G G G G G G G . G    
653	. . . . . . . . T T T T T T T T T T T T T T T T . T    
654	. . . . . . . . C C C C C C C C C C C C C C C C . C    
655	. . . . . . . . A A A A A A A A A A A A A A A A . A    
656	. . . . . . . . T T T T T T T T T T T T T T T T . T    
657	. . . . . . . . C C C C C C C C C C C C C C C C . C    
658	. . . . . . . . C C C C C C C C C C C C C C C C . C    
659	. . . . . . . . A A A A A A A A A A A A A A A A . A    
660	. . . . . . . . C C C C C C C C C C C C C C C C . C    
661	. . . . . . . . A A A A A A A A A A A A A A A A . A    
662	. . . . . . . . G G G G G G G G G G G G G G G G . G    
663	. . . . . . . . . G G G G G G G G G G G G G G G . G    
664	. . . . . . . . . G G G G G G G G G G G G G G G . G    
665	. . . . . . . . . G G G G G G G G G G G G G G G . G    
666	. . . . . . . . . A A A A A A A A A A A A A A A . A    
667	. . . . . . . . . T T T T T T T T T T T T T T T . T    
668	. . . . . . . . . A A A A A A A A A A A A A A A . A    
669	. . . . . . . . . A A A A A A A A A A A A A A A . A    
670	. . . . . . . . . T T T T T T T T T T T T T T T . T    
671	. . . . . . . . . G G G G G G G G G G G G G G G . G    
672	. . . . . . . . . A A A A A A A A A A A A A A A . A    
673	. . . . . . . . . A A A A A A A A A A A A A A A . A    
674	. . . . . . . . . A A A A A A A A A A A A A A A . A    
675	. . . . . . . . . C A C C C C C C C C C C C C C . C    
676	. . . . . . . . . G . . G G G G G G G G G G G G . G    
677	. . . . . . . . . G . . G G G G G G G G G G G G . G    
678	. . . . . . . . . A . . A A A A A A A A A A A A . A    
679	. . . . . . . . . C . . C C C C C C C C C C C C . C    
680	. . . . . . . . . G . . G G G G G G G G G G G G . G    
681	. . . . . . . . . T . . T T T T T T T T T T T T . T    
682	. . . . . . . . . C . . C C C C C C C C C C C C . C    
683	. . . . . . . . . A . . A A A A A A A A A A A A . A    
684	. . . . . . . . . A . . A A A A A A A A A A A A . A    
685	. . . . . . . . . C . . C C C C C C C C C C C C . C    
686	. . . . . . . . . A . . A A A A A A A A A A A A . A    
687	. . . . . . . . . A . . A A A A A A A A A A A A . A    
688	. . . . . . . . . T . . T T T T T T T T T T T T . T    
689	. . . . . . . . . T . . T T T T T T T T T T T T . T    
690	. . . . . . . . . T . . T T T T T T T T T T T T . T    
691	. . . . . . . . . C . . C C C C C C C C C C C C . C    
692	. . . . . . . . . A . . A A A A A A A A A A A A . A    
693	. . . . . . . . . T . . T T T T T T T T T T T T . T    
694	. . . . . . . . . T . . T T T T T T T T T T T T . T    
695	. . . . . . . . . T . . T T T T T T T T T T T T . T    
696	. . . . . . . . . A . . A A A A A A A A A A A A . A    
697	. . . . . . . . . G . . G G G G G G G G G G G G . G    
698	. . . . . . . . . C . . A A A A A A A A A A A A . A    
699	. . . . . . . . . . . . C C C C C C C C C C C C . C    
700	. . . . . . . . . . . . A A A A A A A A A A A A . A    
701	. . . . . . . . . . . . A A A A A A A A A A A A . A    
702	. . . . . . . . . . . . T T T T T T T T T T T T . T    
703	. . . . . . . . . . . . A A A A A A A A A A A A . A    
704	. . . . . . . . . . . . T T T T T T T T T T T T . T    
705	. . . . . . . . . . . . A A A A A A A A A A A A . A    
706	. . . . . . . . . . . . C C C C C C C C C C C C . C    
707	. . . . . . . . . . . . T T T T T T T T T T T T . T    
708	. . . . . . . . . . . . A A A A A A A A A A A A . A    
709	. . . . . . . . . . . . T T T T T T T T T T T T . T    
710	. . . . . . . . . . . . A A A A A A A A A A A A . A    
711	. . . . . . . . . . . . G G G G G G G G G G G G . G    
712	. . . . . . . . . . . . T T T T T T T T T T T T . T    
713	. . . . . . . . . . . . A A A A A A A A A A A A . A    
714	. . . . . . . . . . . . G G G G G G G G G G G G . G    
715	. . . . . . . . . . . . A A A A A A A A A A A A . A    
716	. . . . . . . . . . . . C C C C C C C C C C C C . C    
717	. . . . . . . . . . . . T T T T T T T T T T T T . T    
718	. . . . . . . . . . . . A A A A A A A A A A A A . A    
719	. . . . . . . . . . . . A A A A A A A A A A A A . A    
720	. . . . . . . . . . . . T T T T T T T T T T T T . T    
721	. . . . . . . . . . . . A A A A A A A A A A A A . A    
722	. . . . . . . . . . . . A A A A A A A A A A A A . A    
723	. . . . . . . . . . . . T T T T T T T T T T T T . T    
724	. . . . . . . . . . . . A A A A A A A A A A A A . A    
725	. . . . . . . . . . . . G T G G G G G G G G G G . G    
726	. . . . . . . . . . . . T . T T T T T T T T T T . T    
727	. . . . . . . . . . . . T . T T T T T T T T T T . T    
728	. . . . . . . . . . . . A . T A A A A A A A A A . A    
729	. . . . . . . . . . . . C . . C C C C C C C C C . C    
730	. . . . . . . . . . . . T . . T T T T T T T T T . T    
731	. . . . . . . . . . . . A . . A A A A A A A A A . A    
732	. . . . . . . . . . . . A . . A A A A A A A A A . A    
733	. . . . . . . . . . . . A . . A A A A A A A A A . A    
734	. . . . . . . . . . . . A . . A A A A A A A A A . A    
735	. . . . . . . . . . . . T . . T T T T T T T T T . T    
736	. . . . . . . . . . . . C . . C C C C C C C C C . C    
737	. . . . . . . . . . . . T . . T T T T T T T T T . T    
738	. . . . . . . . . . . . A . . A A A A A A A A A . A    
739	. . . . . . . . . . . . A . . A A A A A A A A A . A    
740	. . . . . . . . . . . . T . . T T T T T T T T T . T    
741	. . . . . . . . . . . . A . . A A A A A A A A A . A    
742	. . . . . . . . . . . . T . . T T T T T T T T T . T    
743	. . . . . . . . . . . . A . . A A A A A A A A A . A    
744	. . . . . . . . . . . . A . . A A A A A A A A A . A    
745	. . . . . . . . . . . . G . . G G G G G G G G G . G    
746	. . . . . . . . . . . . C . . C C C C C C C C C . C    
747	. . . . . . . . . . . . A . . A A A A A A A A A . A    
748	. . . . . . . . . . . . C . . C C C C C C C C C . C    
749	. . . . . . . . . . . . T . . T T T T T T T T T . T    
750	. . . . . . . . . . . . A . . A A A A A A A A A . A    
751	. . . . . . . . . . . . G . . G G G G G G G G G . G    
752	. . . . . . . . . . . . T . . T T T T T T T T T . T    
753	. . . . . . . . . . . . C . . C C C C C C C C C . C    
754	. . . . . . . . . . . . T . . T T T T T T T T T . T    
755	. . . . . . . . . . . . A . . A A A A A A A A A . A    
756	. . . . . . . . . . . . C . . C C C C C C C C C . C    
757	. . . . . . . . . . . . A . . A A A A A A A A A . A    
758	. . . . . . . . . . . . C . . C C C C C C C C C . C    
759	. . . . . . . . . . . . A . . A A A A A A A A A . A    
760	. . . . . . . . . . . . A . . A C C A A A A A A . A    
761	. . . . . . . . . . . . A . . A . . A A A A A A . A    
762	. . . . . . . . . . . . A . . A . . A A A A A A . A    
763	. . . . . . . . . . . . G . . G . . G G G G G G . G    
764	. . . . . . . . . . . . G . . G . . G G G G G G . G    
765	. . . . . . . . . . . . T . . T . . T T T T T T . T    
766	. . . . . . . . . . . . T . . T . . T T T T T T . T    
767	. . . . . . . . . . . . T . . T . . T T T T T T . T    
768	. . . . . . . . . . . . A . . A . . A A A A A A . A    
769	. . . . . . . . . . . . C . . C . . C C C C C C . C    
770	. . . . . . . . . . . . C . . G . . G G G G G G . G    
771	. . . . . . . . . . . . . . . A . . A A A A A A . A    
772	. . . . . . . . . . . . . . . A . . A A A A A A . A    
773	. . . . . . . . . . . . . . . G . . G G G G G G . G    
774	. . . . . . . . . . . . . . . T . . T T T T T T . T    
775	. . . . . . . . . . . . . . . G . . G G G G G G . G    
776	. . . . . . . . . . . . . . . C . . A A A A A A . A    
777	. . . . . . . . . . . . . . . . . . C C C C C C . C    
778	. . . . . . . . . . . . . . . . . . A A A A A A . A    
779	. . . . . . . . . . . . . . . . . . G G G G G G . G    
780	. . . . . . . . . . . . . . . . . . A A A A A A . A    
781	. . . . . . . . . . . . . . . . . . T T T T T T . T    
782	. . . . . . . . . . . . . . . . . . T T T T T T . T    
783	. . . . . . . . . . . . . . . . . . C C C C C C . C    
784	. . . . . . . . . . . . . . . . . . A A A A A A . A    
785	. . . . . . . . . . . . . . . . . . G G G G G G . G    
786	. . . . . . . . . . . . . . . . . . C C C C C C . C    
787	. . . . . . . . . . . . . . . . . . C C C C C C . C    
788	. . . . . . . . . . . . . . . . . . G G G G G G . G    
789	. . . . . . . . . . . . . . . . . . C C C C C C . C    
790	. . . . . . . . . . . . . . . . . . A A A A A A . A    
791	. . . . . . . . . . . . . . . . . . G G G G G G . G    
792	. . . . . . . . . . . . . . . . . . G G G G G G . G    
793	. . . . . . . . . . . . . . . . . . T T T T T T . T    
794	. . . . . . . . . . . . . . . . . . A A A A A A . A    
795	. . . . . . . . . . . . . . . . . . G G G G G G . G    
796	. . . . . . . . . . . . . . . . . . G G G G G G . G    
797	. . . . . . . . . . . . . . . . . . G G G G G G . G    
798	. . . . . . . . . . . . . . . . . . G G G G G G . G    
799	. . . . . . . . . . . . . . . . . . C C C C C C . C    
800	. . . . . . . . . . . . . . . . . . C C C C C C . C    
801	. . . . . . . . . . . . . . . . . . A A A A A A . A    
802	. . . . . . . . . . . . . . . . . . A A A A A A . A    
803	. . . . . . . . . . . . . . . . . . T T T T T T . T    
804	. . . . . . . . . . . . . . . . . . C C C C C C . C    
805	. . . . . . . . . . . . . . . . . . A A A A A A . A    
806	. . . . . . . . . . . . . . . . . . G G G G G G . G    
807	. . . . . . . . . . . . . . . . . . G G G G G G . G    
808	. . . . . . . . . . . . . . . . . . G G G G G G . G    
809	. . . . . . . . . . . . . . . . . . A A A A A A . A    
810	. . . . . . . . . . . . . . . . . . G G G G G G . G    
811	. . . . . . . . . . . . . . . . . . C C C C C C . C    
812	. . . . . . . . . . . . . . . . . . C C C C C C . C    
813	. . . . . . . . . . . . . . . . . . C C C C C C . C    
814	. . . . . . . . . . . . . . . . . . A A A A A A A A    
815	. . . . . . . . . . . . . . . . . . G G G G G G G G    
816	. . . . . . . . . . . . . . . . . . T T T T T T T T    
817	. . . . . . . . . . . . . . . . . . G G G G G G G G    
818	. . . . . . . . . . . . . . . . . . G G G G G G G G    
819	. . . . . . . . . . . . . . . . . . A A A A A A A A    
820	. . . . . . . . . . . . . . . . . . T T T T T T T T    
821	. . . . . . . . . . . . . . . . . . G G G G G G G G    
822	. . . . . . . . . . . . . . . . . . T T T T T T T T    
823	. . . . . . . . . . . . . . . . . . C C C C C C C C    
824	. . . . . . . . . . . . . . . . . . G G G G G G G G    
825	. . . . . . . . . . . . . . . . . . C C C C C C C C    
826	. . . . . . . . . . . . . . . . . . G G G G G G G G    
827	. . . . . . . . . . . . . . . . . . C C C C C C C C    
828	. . . . . . . . . . . . . . . . . . A A A A A A A A    
829	. . . . . . . . . . . . . . . . . . C C C C C C C C    
830	. . . . . . . . . . . . . . . . . . A A A A A A A A    
831	. . . . . . . . . . . . . . . . . . C C C C C C C C    
832	. . . . . . . . . . . . . . . . . . T T T T T T T T    
833	. . . . . . . . . . . . . . . . . . C C C C C C C C    
834	. . . . . . . . . . . . . . . . . . T T T T T T T T    
835	. . . . . . . . . . . . . . . . . . G G G G G G G G    
836	. . . . . . . . . . . . . . . . . . C G G G G G G G    
837	. . . . . . . . . . . . . . . . . . . . A A A A A A    
838	. . . . . . . . . . . . . . . . . . . . G G G G G G    
839	. . . . . . . . . . . . . . . . . . . . G G G G G G    
840	. . . . . . . . . . . . . . . . . . . . A A A A A A    
841	. . . . . . . . . . . . . . . . . . . . A A A A A A    
842	. . . . . . . . . . . . . . . . . . . . G G G G G G    
843	. . . . . . . . . . . . . . . . . . . . G G G G G G    
844	. . . . . . . . . . . . . . . . . . . . A A A A A A    
845	. . . . . . . . . . . . . . . . . . . . T T T T T T    
846	. . . . . . . . . . . . . . . . . . . . T T T T T T    
847	. . . . . . . . . . . . . . . . . . . . G G G G G G    
848	. . . . . . . . . . . . . . . . . . . . G G G G G G    
849	. . . . . . . . . . . . . . . . . . . . G G G G G G    
850	. . . . . . . . . . . . . . . . . . . . G G G G G G    
851	. . . . . . . . . . . . . . . . . . . . G G G G G G    
852	. . . . . . . . . . . . . . . . . . . . A A A A A A    
853	. . . . . . . . . . . . . . . . . . . . T T T T T T    
854	. . . . . . . . . . . . . . . . . . . . G G G G G G    
855	. . . . . . . . . . . . . . . . . . . . G G G G G G    
856	. . . . . . . . . . . . . . . . . . . . G G G G G G    
857	. . . . . . . . . . . . . . . . . . . . C C C C C C    
858	. . . . . . . . . . . . . . . . . . . . G G G G G G    
859	. . . . . . . . . . . . . . . . . . . . G G G G G G    
860	. . . . . . . . . . . . . . . . . . . . G G G G G G    
861	. . . . . . . . . . . . . . . . . . . . C C C C C C    
862	. . . . . . . . . . . . . . . . . . . . C C C C C C    
863	. . . . . . . . . . . . . . . . . . . . T T T T T T    
864	. . . . . . . . . . . . . . . . . . . . T T T T T T    
865	. . . . . . . . . . . . . . . . . . . . G G G G G G    
866	. . . . . . . . . . . . . . . . . . . . T T T T T T    
867	. . . . . . . . . . . . . . . . . . . . C C C C C C    
868	. . . . . . . . . . . . . . . . . . . . T T T T T T    
869	. . . . . . . . . . . . . . . . . . . . T T T T T T    
870	. . . . . . . . . . . . . . . . . . . . T T T T T T    
871	. . . . . . . . . . . . . . . . . . . . A A A A A A    
872	. . . . . . . . . . . . . . . . . . . . G G G G G G    
873	. . . . . . . . . . . . . . . . . . . . A A A A A A    
874	. . . . . . . . . . . . . . . . . . . . T T T T T T    
875	. . . . . . . . . . . . . . . . . . . . A A A A A A    
876	. . . . . . . . . . . . . . . . . . . . A A A A A A    
877	. . . . . . . . . . . . . . . . . . . . C C C C C C    
878	. . . . . . . . . . . . . . . . . . . . C C C C C C    
879	. . . . . . . . . . . . . . . . . . . . T T T T T T    
880	. . . . . . . . . . . . . . . . . . . . C C C C C C    
881	. . . . . . . . . . . . . . . . . . . . A A A A A A    
882	. . . . . . . . . . . . . . . . . . . . A A A A A A    
883	. . . . . . . . . . . . . . . . . . . . C C C C C C    
884	. . . . . . . . . . . . . . . . . . . . G G G G G G    
885	. . . . . . . . . . . . . . . . . . . . T T T T T T    
886	. . . . . . . . . . . . . . . . . . . . A A A A A A    
887	. . . . . . . . . . . . . . . . . . . . A A A A A A    
888	. . . . . . . . . . . . . . . . . . . . T T T T T T    
889	. . . . . . . . . . . . . . . . . . . . T T T T T T    
890	. . . . . . . . . . . . . . . . . . . . G G G G G G    
891	. . . . . . . . . . . . . . . . . . . . G G G G G G    
892	. . . . . . . . . . . . . . . . . . . . C C C C C C    
893	. . . . . . . . . . . . . . . . . . . . C C C C C C    
894	. . . . . . . . . . . . . . . . . . . . G G G G G G    
895	. . . . . . . . . . . . . . . . . . . . G G G G G G    
896	. . . . . . . . . . . . . . . . . . . . C C C C C C    
897	. . . . . . . . . . . . . . . . . . . . A A A A A A    
898	. . . . . . . . . . . . . . . . . . . . G G G G G G    
899	. . . . . . . . . . . . . . . . . . . . C C C C C C    
900	. . . . . . . . . . . . . . . . . . . . A A A A A A    
901	. . . . . . . . . . . . . . . . . . . . T T T T T T    
902	. . . . . . . . . . . . . . . . . . . . G G G G G G    
903	. . . . . . . . . . . . . . . . . . . . C C C C C C    
904	. . . . . . . . . . . . . . . . . . . . C C C C C C    
905	. . . . . . . . . . . . . . . . . . . . A A A A A A    
906	. . . . . . . . . . . . . . . . . . . . G G G G G G    
907	. . . . . . . . . . . . . . . . . . . . T T T T T T    
908	. . . . . . . . . . . . . . . . . . . . C C C C C C    
909	. . . . . . . . . . . . . . . . . . . . T T T T T T    
910	. . . . . . . . . . . . . . . . . . . . T T T T T T    
911	. . . . . . . . . . . . . . . . . . . . G G G G G G    
912	. . . . . . . . . . . . . . . . . . . . C C C C C C    
913	. . . . . . . . . . . . . . . . . . . . C C C C C C    
914	. . . . . . . . . . . . . . . . . . . . T T T T T T    
915	. . . . . . . . . . . . . . . . . . . . G G G G G G    
916	. . . . . . . . . . . . . . . . . . . . A A A A A A    
917	. . . . . . . . . . . . . . . . . . . . G G G G G G    
918	. . . . . . . . . . . . . . . . . . . . C C C C C C    
919	. . . . . . . . . . . . . . . . . . . . G G G G G G    
920	. . . . . . . . . . . . . . . . . . . . C C C C C C    
921	. . . . . . . . . . . . . . . . . . . . T T T T T T    
922	. . . . . . . . . . . . . . . . . . . . G G G G G G    
923	. . . . . . . . . . . . . . . . . . . . C C C C C C    
924	. . . . . . . . . . . . . . . . . . . . A A A A A A    
925	. . . . . . . . . . . . . . . . . . . . C C C C C C    
926	. . . . . . . . . . . . . . . . . . . . G G G G G G    
927	. . . . . . . . . . . . . . . . . . . . G G A G G G    
928	. . . . . . . . . . . . . . . . . . . . T T T T T T    
929	. . . . . . . . . . . . . . . . . . . . G G G G G G    
930	. . . . . . . . . . . . . . . . . . . . C C C C C C    
931	. . . . . . . . . . . . . . . . . . . . C C C C C C    
932	. . . . . . . . . . . . . . . . . . . . G G G G G G    
933	. . . . . . . . . . . . . . . . . . . . T T T T T T    
934	. . . . . . . . . . . . . . . . . . . . A A A A A A    
935	. . . . . . . . . . . . . . . . . . . . C C C C C C    
936	. . . . . . . . . . . . . . . . . . . . A A A A A A    
937	. . . . . . . . . . . . . . . . . . . . T T T T T T    
938	. . . . . . . . . . . . . . . . . . . . C C C C C C    
939	. . . . . . . . . . . . . . . . . . . . C C C C C C    
940	. . . . . . . . . . . . . . . . . . . . A A A A A A    
941	. . . . . . . . . . . . . . . . . . . . A A A A A A    
942	. . . . . . . . . . . . . . . . . . . . C C C C C C    
943	. . . . . . . . . . . . . . . . . . . . C C C C C C    
944	. . . . . . . . . . . . . . . . . . . . T T T T T T    
945	. . . . . . . . . . . . . . . . . . . . T T T T T T    
946	. . . . . . . . . . . . . . . . . . . . C C C C C C    
947	. . . . . . . . . . . . . . . . . . . . A A A A A A    
948	. . . . . . . . . . . . . . . . . . . . T T T T T T    
949	. . . . . . . . . . . . . . . . . . . . C C C C C C    
950	. . . . . . . . . . . . . . . . . . . . G G G G G G    
951	. . . . . . . . . . . . . . . . . . . . A A A A A A    
952	. . . . . . . . . . . . . . . . . . . . T T T T T T    
953	. . . . . . . . . . . . . . . . . . . . G G G G G G    
954	. . . . . . . . . . . . . . . . . . . . G G G G G G    
955	. . . . . . . . . . . . . . . . . . . . C C C C C C    
956	. . . . . . . . . . . . . . . . . . . . C C C C C C    
957	. . . . . . . . . . . . . . . . . . . . T T T T T T    
958	. . . . . . . . . . . . . . . . . . . . G G G G G G    
959	. . . . . . . . . . . . . . . . . . . . C C C C C C    
960	. . . . . . . . . . . . . . . . . . . . A A A A A A    
961	. . . . . . . . . . . . . . . . . . . . C C C C C C    
962	. . . . . . . . . . . . . . . . . . . . G G G G G G    
963	. . . . . . . . . . . . . . . . . . . . T T T T T T    
964	. . . . . . . . . . . . . . . . . . . . T T T T T T    
965	. . . . . . . . . . . . . . . . . . . . T T T T T T    
966	. . . . . . . . . . . . . . . . . . . . A A A A A A    
967	. . . . . . . . . . . . . . . . . . . . C C C C C C    
968	. . . . . . . . . . . . . . . . . . . . G G G G G G    
969	. . . . . . . . . . . . . . . . . . . . A A A A A A    
970	. . . . . . . . . . . . . . . . . . . . T T T T T T    
971	. . . . . . . . . . . . . . . . . . . . G G G G G G    
972	. . . . . . . . . . . . . . . . . . . . A A A A A A    
973	. . . . . . . . . . . . . . . . . . . . C C C C C C    
974	. . . . . . . . . . . . . . . . . . . . A A A A A A    
975	. . . . . . . . . . . . . . . . . . . . T T T T T T    
976	. . . . . . . . . . . . . . . . . . . . C C C C C C    
977	. . . . . . . . . . . . . . . . . . . . A A A A A A    
978	. . . . . . . . . . . . . . . . . . . . C C C C C C    
979	. . . . . . . . . . . . . . . . . . . . C C C C C C    
980	. . . . . . . . . . . . . . . . . . . . G G G G G G    
981	. . . . . . . . . . . . . . . . . . . . T T T T T T    
982	. . . . . . . . . . . . . . . . . . . . C C C C C C    
983	. . . . . . . . . . . . . . . . . . . . C C C C C C    
984	. . . . . . . . . . . . . . . . . . . . T T T T T T    
985	. . . . . . . . . . . . . . . . . . . . T T T T T T    
986	. . . . . . . . . . . . . . . . . . . . A A A A A A    
987	. . . . . . . . . . . . . . . . . . . . A A A A A A    
988	. . . . . . . . . . . . . . . . . . . . A A A A A A    
989	. . . . . . . . . . . . . . . . . . . . G G G G G G    
990	. . . . . . . . . . . . . . . . . . . . G G G G G G    
991	. . . . . . . . . . . . . . . . . . . . A A A A A A    
992	. . . . . . . . . . . . . . . . . . . . C C C C C C    
993	. . . . . . . . . . . . . . . . . . . . A A A A A A    
994	. . . . . . . . . . . . . . . . . . . . C C C C C C    
995	. . . . . . . . . . . . . . . . . . . . C C C C C C    
996	. . . . . . . . . . . . . . . . . . . . T T T T T T    
997	. . . . . . . . . . . . . . . . . . . . C C C C C C    
998	. . . . . . . . . . . . . . . . . . . . G G G G G G    
999	. . . . . . . . . . . . . . . . . . . . T T T T T T    
1000	. . . . . . . . . . . . . . . . . . . . C C C C C C    
1001	. . . . . . . . . . . . . . . . . . . . A A A A A A    
1002	. . . . . . . . . . . . . . . . . . . . C C C C C C    
1003	. . . . . . . . . . . . . . . . . . . . T T T T T T    
1004	. . . . . . . . . . . . . . . . . . . . G G G G G G    
1005	. . . . . . . . . . . . . . . . . . . . T T T T T T    
1006	. . . . . . . . . . . . . . . . . . . . C C C C C C    
1007	. . . . . . . . . . . . . . . . . . . . G G G G G G    
1008	. . . . . . . . . . . . . . . . . . . . G G G G G G    
1009	. . . . . . . . . . . . . . . . . . . . C C C C C C    
1010	. . . . . . . . . . . . . . . . . . . . A A A A A A    
1011	. . . . . . . . . . . . . . . . . . . . G G G G G G    
1012	. . . . . . . . . . . . . . . . . . . . A A A A A A    
1013	. . . . . . . . . . . . . . . . . . . . G G G G G G    
1014	. . . . . . . . . . . . . . . . . . . . G G G G G G    
1015	. . . . . . . . . . . . . . . . . . . . A A A A A A    
1016	. . . . . . . . . . . . . . . . . . . . G G G G G G    
1017	. . . . . . . . . . . . . . . . . . . . T T T T T T    
1018	. . . . . . . . . . . . . . . . . . . . A A A A A A    
1019	. . . . . . . . . . . . . . . . . . . . G G G G G G    
1020	. . . . . . . . . . . . . . . . . . . . T T T T T T    
1021	. . . . . . . . . . . . . . . . . . . . C C C C C C    
1022	. . . . . . . . . . . . . . . . . . . . A A A A A A    
1023	. . . . . . . . . . . . . . . . . . . . T T T T T T    
1024	. . . . . . . . . . . . . . . . . . . . A A A A A A    
1025	. . . . . . . . . . . . . . . . . . . . C C C C C C    
1026	. . . . . . . . . . . . . . . . . . . . A A A A A A    
1027	. . . . . . . . . . . . . . . . . . . . T T T T T T    
1028	. . . . . . . . . . . . . . . . . . . . G G G G G G    
1029	. . . . . . . . . . . . . . . . . . . . G G G G G G    
1030	. . . . . . . . . . . . . . . . . . . . C C C C C C    
1031	. . . . . . . . . . . . . . . . . . . . C A A A A A    
1032	. . . . . . . . . . . . . . . . . . . . . C C C C C    
1033	. . . . . . . . . . . . . . . . . . . . . G G G G G    
1034	. . . . . . . . . . . . . . . . . . . . . C C C C C    
1035	. . . . . . . . . . . . . . . . . . . . . A A A A A    
1036	. . . . . . . . . . . . . . . . . . . . . C C C C C    
1037	. . . . . . . . . . . . . . . . . . . . . C C C C C    
1038	. . . . . . . . . . . . . . . . . . . . . G G G G G    
1039	. . . . . . . . . . . . . . . . . . . . . T T T T T    
1040	. . . . . . . . . . . . . . . . . . . . . T T T T T    
1041	. . . . . . . . . . . . . . . . . . . . . T T T T T    
1042	. . . . . . . . . . . . . . . . . . . . . T T T T T    
1043	. . . . . . . . . . . . . . . . . . . . . C C C C C    
1044	. . . . . . . . . . . . . . . . . . . . . C C C C C    
1045	. . . . . . . . . . . . . . . . . . . . . C C C C C    
1046	. . . . . . . . . . . . . . . . . . . . . G G G G G    
1047	. . . . . . . . . . . . . . . . . . . . . G G G G G    
1048	. . . . . . . . . . . . . . . . . . . . . T T T T T    
1049	. . . . . . . . . . . . . . . . . . . . . G G G G G    
1050	. . . . . . . . . . . . . . . . . . . . . G G G G G    
1051	. . . . . . . . . . . . . . . . . . . . . T T T T T    
1052	. . . . . . . . . . . . . . . . . . . . . G G G G G    
1053	. . . . . . . . . . . . . . . . . . . . . G G G G G    
1054	. . . . . . . . . . . . . . . . . . . . . A A A A A    
1055	. . . . . . . . . . . . . . . . . . . . . G G G G G    
1056	. . . . . . . . . . . . . . . . . . . . . G G G G G    
1057	. . . . . . . . . . . . . . . . . . . . . G G G G G    
1058	. . . . . . . . . . . . . . . . . . . . . G G G G G    
1059	. . . . . . . . . . . . . . . . . . . . . T T T T T    
1060	. . . . . . . . . . . . . . . . . . . . . G G G G G    
1061	. . . . . . . . . . . . . . . . . . . . . G G G G G    
1062	. . . . . . . . . . . . . . . . . . . . . G G G G G    
1063	. . . . . . . . . . . . . . . . . . . . . A A A A A    
1064	. . . . . . . . . . . . . . . . . . . . . G G G G G    
1065	. . . . . . . . . . . . . . . . . . . . . G G G G G    
1066	. . . . . . . . . . . . . . . . . . . . . G G G G G    
1067	. . . . . . . . . . . . . . . . . . . . . G G G G G    
1068	. . . . . . . . . . . . . . . . . . . . . A A A A A    
1069	. . . . . . . . . . . . . . . . . . . . . C C C C C    
1070	. . . . . . . . . . . . . . . . . . . . . A A A A A    
1071	. . . . . . . . . . . . . . . . . . . . . G G G G G    
1072	. . . . . . . . . . . . . . . . . . . . . A A A A A    
1073	. . . . . . . . . . . . . . . . . . . . . N G G G G    
1074	. . . . . . . . . . . . . . . . . . . . . . G G G G    
1075	. . . . . . . . . . . . . . . . . . . . . . A A A A    
1076	. . . . . . . . . . . . . . . . . . . . . . C C C C    
1077	. . . . . . . . . . . . . . . . . . . . . . A A A A    
1078	. . . . . . . . . . . . . . . . . . . . . . C C C C    
1079	. . . . . . . . . . . . . . . . . . . . . . G G G G    
1080	. . . . . . . . . . . . . . . . . . . . . . G G G G    
1081	. . . . . . . . . . . . . . . . . . . . . . T T T T    
1082	. . . . . . . . . . . . . . . . . . . . . . G G G G    
1083	. . . . . . . . . . . . . . . . . . . . . . G G G G    
1084	. . . . . . . . . . . . . . . . . . . . . . T T T T    
1085	. . . . . . . . . . . . . . . . . . . . . . C C C C    
1086	. . . . . . . . . . . . . . . . . . . . . . T T T T    
1087	. . . . . . . . . . . . . . . . . . . . . . C G G G    
1088	. . . . . . . . . . . . . . . . . . . . . . . C C C    
1089	. . . . . . . . . . . . . . . . . . . . . . . T T T    
1090	. . . . . . . . . . . . . . . . . . . . . . . G G G    
1091	. . . . . . . . . . . . . . . . . . . . . . . G G G    
1092	. . . . . . . . . . . . . . . . . . . . . . . G G G    
1093	. . . . . . . . . . . . . . . . . . . . . . . T T T    
1094	. . . . . . . . . . . . . . . . . . . . . . . G G G    
1095	. . . . . . . . . . . . . . . . . . . . . . . G G G    
1096	. . . . . . . . . . . . . . . . . . . . . . . C C C    
1097	. . . . . . . . . . . . . . . . . . . . . . . T T T    
1098	. . . . . . . . . . . . . . . . . . . . . . . A A A    
1099	. . . . . . . . . . . . . . . . . . . . . . . C C C    
1100	. . . . . . . . . . . . . . . . . . . . . . . A A A    
1101	. . . . . . . . . . . . . . . . . . . . . . . G G G    
1102	. . . . . . . . . . . . . . . . . . . . . . . A A A    
1103	. . . . . . . . . . . . . . . . . . . . . . . G G G    
1104	. . . . . . . . . . . . . . . . . . . . . . . G G G    
1105	. . . . . . . . . . . . . . . . . . . . . . . A A A    
1106	. . . . . . . . . . . . . . . . . . . . . . . A A A    
1107	. . . . . . . . . . . . . . . . . . . . . . . G G G    
1108	. . . . . . . . . . . . . . . . . . . . . . . A A A    
1109	. . . . . . . . . . . . . . . . . . . . . . . C C C    
1110	. . . . . . . . . . . . . . . . . . . . . . . C C C    
1111	. . . . . . . . . . . . . . . . . . . . . . . T T T    
1112	. . . . . . . . . . . . . . . . . . . . . . . C C C    
1113	. . . . . . . . . . . . . . . . . . . . . . . T T T    
1114	. . . . . . . . . . . . . . . . . . . . . . . G G G    
1115	. . . . . . . . . . . . . . . . . . . . . . . C C C    
1116	. . . . . . . . . . . . . . . . . . . . . . . T T T    
1117	. . . . . . . . . . . . . . . . . . . . . . . G G G    
1118	. . . . . . . . . . . . . . . . . . . . . . . C C C    
1119	. . . . . . . . . . . . . . . . . . . . . . . A A A    
1120	. . . . . . . . . . . . . . . . . . . . . . . G G G    
1121	. . . . . . . . . . . . . . . . . . . . . . . C C C    
1122	. . . . . . . . . . . . . . . . . . . . . . . T T T    
1123	. . . . . . . . . . . . . . . . . . . . . . . C C C    
1124	. . . . . . . . . . . . . . . . . . . . . . . C C C    
1125	. . . . . . . . . . . . . . . . . . . . . . . A A A    
1126	. . . . . . . . . . . . . . . . . . . . . . . C C C    
1127	. . . . . . . . . . . . . . . . . . . . . . . G G G    
1128	. . . . . . . . . . . . . . . . . . . . . . . G G G    
1129	. . . . . . . . . . . . . . . . . . . . . . . T T T    
1130	. . . . . . . . . . . . . . . . . . . . . . . C C C    
1131	. . . . . . . . . . . . . . . . . . . . . . . T T T    
1132	. . . . . . . . . . . . . . . . . . . . . . . T T T    
1133	. . . . . . . . . . . . . . . . . . . . . . . G G G    
1134	. . . . . . . . . . . . . . . . . . . . . . . G G G    
1135	. . . . . . . . . . . . . . . . . . . . . . . T T T    
1136	. . . . . . . . . . . . . . . . . . . . . . . G G G    
1137	. . . . . . . . . . . . . . . . . . . . . . . T T T    
1138	. . . . . . . . . . . . . . . . . . . . . . . G G G    
1139	. . . . . . . . . . . . . . . . . . . . . . . T T T    
1140	. . . . . . . . . . . . . . . . . . . . . . . G G G    
1141	. . . . . . . . . . . . . . . . . . . . . . . T T T    
1142	. . . . . . . . . . . . . . . . . . . . . . . G G G    
1143	. . . . . . . . . . . . . . . . . . . . . . . T T T    
1144	. . . . . . . . . . . . . . . . . . . . . . . A A A    
1145	. . . . . . . . . . . . . . . . . . . . . . . G G G    
1146	. . . . . . . . . . . . . . . . . . . . . . . C C C    
1147	. . . . . . . . . . . . . . . . . . . . . . . C C C    
1148	. . . . . . . . . . . . . . . . . . . . . . . C C C    
1149	. . . . . . . . . . . . . . . . . . . . . . . A A A    
1150	. . . . . . . . . . . . . . . . . . . . . . . T T T    
1151	. . . . . . . . . . . . . . . . . . . . . . . A A A    
1152	. . . . . . . . . . . . . . . . . . . . . . . G G G    
1153	. . . . . . . . . . . . . . . . . . . . . . . A A A    
1154	. . . . . . . . . . . . . . . . . . . . . . . G G G    
1155	. . . . . . . . . . . . . . . . . . . . . . . G G G    
1156	. . . . . . . . . . . . . . . . . . . . . . . G G G    
1157	. . . . . . . . . . . . . . . . . . . . . . . G G G    
1158	. . . . . . . . . . . . . . . . . . . . . . . A A A    
1159	. . . . . . . . . . . . . . . . . . . . . . . G G G    
1160	. . . . . . . . . . . . . . . . . . . . . . . A A A    
1161	. . . . . . . . . . . . . . . . . . . . . . . G G G    
1162	. . . . . . . . . . . . . . . . . . . . . . . T T T    
1163	. . . . . . . . . . . . . . . . . . . . . . . G G G    
1164	. . . . . . . . . . . . . . . . . . . . . . . A A A    
1165	. . . . . . . . . . . . . . . . . . . . . . . A A A    
1166	. . . . . . . . . . . . . . . . . . . . . . . G G G    
1167	. . . . . . . . . . . . . . . . . . . . . . . C C C    
1168	. . . . . . . . . . . . . . . . . . . . . . . C C C    
1169	. . . . . . . . . . . . . . . . . . . . . . . G G G    
1170	. . . . . . . . . . . . . . . . . . . . . . . T T T    
1171	. . . . . . . . . . . . . . . . . . . . . . . T T T    
1172	. . . . . . . . . . . . . . . . . . . . . . . G G G    
1173	. . . . . . . . . . . . . . . . . . . . . . . C C C    
1174	. . . . . . . . . . . . . . . . . . . . . . . T T T    
1175	. . . . . . . . . . . . . . . . . . . . . . . G G G    
1176	. . . . . . . . . . . . . . . . . . . . . . . T T T    
1177	. . . . . . . . . . . . . . . . . . . . . . . A A A    
1178	. . . . . . . . . . . . . . . . . . . . . . . C C C    
1179	. . . . . . . . . . . . . . . . . . . . . . . A A A    
1180	. . . . . . . . . . . . . . . . . . . . . . . G G G    
1181	. . . . . . . . . . . . . . . . . . . . . . . C C C    
1182	. . . . . . . . . . . . . . . . . . . . . . . G G G    
1183	. . . . . . . . . . . . . . . . . . . . . . . A A A    
1184	. . . . . . . . . . . . . . . . . . . . . . . C C C    
1185	. . . . . . . . . . . . . . . . . . . . . . . A A A    
1186	. . . . . . . . . . . . . . . . . . . . . . . T T T    
1187	. . . . . . . . . . . . . . . . . . . . . . . G G G    
1188	. . . . . . . . . . . . . . . . . . . . . . . T T T    
1189	. . . . . . . . . . . . . . . . . . . . . . . G G G    
1190	. . . . . . . . . . . . . . . . . . . . . . . G G G    
1191	. . . . . . . . . . . . . . . . . . . . . . . T T T    
1192	. . . . . . . . . . . . . . . . . . . . . . . G G G    
1193	. . . . . . . . . . . . . . . . . . . . . . . T T T    
1194	. . . . . . . . . . . . . . . . . . . . . . . C C C    
1195	. . . . . . . . . . . . . . . . . . . . . . . C C C    
1196	. . . . . . . . . . . . . . . . . . . . . . . T T T    
1197	. . . . . . . . . . . . . . . . . . . . . . . G G G    
1198	. . . . . . . . . . . . . . . . . . . . . . . G G G    
1199	. . . . . . . . . . . . . . . . . . . . . . . G G G    
1200	. . . . . . . . . . . . . . . . . . . . . . . T T T    
1201	. . . . . . . . . . . . . . . . . . . . . . . C C C    
1202	. . . . . . . . . . . . . . . . . . . . . . . C C C    
1203	. . . . . . . . . . . . . . . . . . . . . . . T T C    
1204	. . . . . . . . . . . . . . . . . . . . . . . C C .    
1205	. . . . . . . . . . . . . . . . . . . . . . . A . .    
1206	. . . . . . . . . . . . . . . . . . . . . . . G . .    
1207	. . . . . . . . . . . . . . . . . . . . . . . A . .    
1208	. . . . . . . . . . . . . . . . . . . . . . . C . .    
1209	. . . . . . . . . . . . . . . . . . . . . . . A . .    
1210	. . . . . . . . . . . . . . . . . . . . . . . C . .    
1211	. . . . . . . . . . . . . . . . . . . . . . . C . .    
1212	. . . . . . . . . . . . . . . . . . . . . . . T . .    
1213	. . . . . . . . . . . . . . . . . . . . . . . C . .    
1214	. . . . . . . . . . . . . . . . . . . . . . . A . .    
1215	. . . . . . . . . . . . . . . . . . . . . . . G . .    
1216	. . . . . . . . . . . . . . . . . . . . . . . A . .    
1217	. . . . . . . . . . . . . . . . . . . . . . . C . .    
1218	. . . . . . . . . . . . . . . . . . . . . . . T . .    
1219	. . . . . . . . . . . . . . . . . . . . . . . T . .    
1220	. . . . . . . . . . . . . . . . . . . . . . . C . .    
1221	. . . . . . . . . . . . . . . . . . . . . . . T . .    
1222	. . . . . . . . . . . . . . . . . . . . . . . C . .    
1223	. . . . . . . . . . . . . . . . . . . . . . . A . .    
1224	. . . . . . . . . . . . . . . . . . . . . . . T . .    
1225	. . . . . . . . . . . . . . . . . . . . . . . C . .    
1226	. . . . . . . . . . . . . . . . . . . . . . . G . .    
1227	. . . . . . . . . . . . . . . . . . . . . . . C . .    
1228	. . . . . . . . . . . . . . . . . . . . . . . C . .    
1229	. . . . . . . . . . . . . . . . . . . . . . . A . .    
1230	. . . . . . . . . . . . . . . . . . . . . . . A . .    
1231	. . . . . . . . . . . . . . . . . . . . . . . C . .    
1232	. . . . . . . . . . . . . . . . . . . . . . . A . .    
1233	. . . . . . . . . . . . . . . . . . . . . . . C . .    
1234	. . . . . . . . . . . . . . . . . . . . . . . C . .    
1235	. . . . . . . . . . . . . . . . . . . . . . . A . .    
1236	. . . . . . . . . . . . . . . . . . . . . . . C . .    
1237	. . . . . . . . . . . . . . . . . . . . . . . C . .    
1238	. . . . . . . . . . . . . . . . . . . . . . . C . .    
1239	. . . . . . . . . . . . . . . . . . . . . . . C . .    
1240	. . . . . . . . . . . . . . . . . . . . . . . C . .
```
